# Global RNA modifications to the MALAT1 triple helix differentially affect thermostability and weaken binding to METTL16

**DOI:** 10.1016/j.jbc.2023.105548

**Published:** 2023-12-11

**Authors:** Mika J. Schievelbein, Carlos Resende, Madeline M. Glennon, Matthew Kerosky, Jessica A. Brown

**Affiliations:** Department of Chemistry and Biochemistry, University of Notre Dame, Notre Dame, Indiana, USA

**Keywords:** long noncoding RNA, MALAT1, METTL16, mRNA, RNA methylation, RNA methyltransferase, RNA modification, RNA–protein interaction, RNA structure, RNA triple helix

## Abstract

Therapeutic mRNAs are generated using modified nucleotides, namely *N*^1^-methylpseudouridine (m^1^Ψ) triphosphate, so that the mRNA evades detection by the immune system. RNA modifications, even at a single-nucleotide position, perturb RNA structure, although it is not well understood how structure and function is impacted by globally modified RNAs. Therefore, we examined the metastasis-associated lung adenocarcinoma transcript 1 triple helix, a highly structured stability element that includes single-, double-, and triple-stranded RNA, globally modified with *N*^6^-methyladenosine (m^6^A), pseudouridine (Ψ), or m^1^Ψ. UV thermal denaturation assays showed that m^6^A destabilizes both the Hoogsteen and Watson–Crick faces of the RNA by ∼20 °C, Ψ stabilizes the Hoogsteen and Watson–Crick faces of the RNA by ∼12 °C, and m^1^Ψ has minimal effect on the stability of the Hoogsteen face of the RNA but increases the stability of the Watson–Crick face by ∼9 °C. Native gel-shift assays revealed that binding of the methyltransferase-like protein 16 to the metastasis-associated lung adenocarcinoma transcript 1 triple helix was weakened by at least 8-, 99-, and 23-fold, respectively, when RNA is globally modified with m^6^A, Ψ, or m^1^Ψ. These results demonstrate that a more thermostable RNA structure does not lead to tighter RNA–protein interactions, thereby highlighting the regulatory power of RNA modifications by multiple means.

The potential for mRNA to function as a therapeutic escalated dramatically upon the discovery that RNA modifications allow *in vitro*-transcribed mRNA to elude detection by the innate immune system (1, 2, 3, 4). Subsequent studies revealed that *N*^1^-methylpseudouridine (m^1^Ψ, Fig. 1*A*) is the preferred RNA modification in therapeutic or exogenous mRNAs due to low immunogenicity and high protein expression (5, 6, 7). Therapeutic mRNA, such as the COVID-19 vaccines manufactured by Moderna and Pfizer/BioNTech, are *in vitro* transcribed using an NTP mixture comprised of ATP, CTP, GTP, and m^1^ΨTP (8). The resulting mRNA products have no unmodified uracil, only m^1^Ψ. Such a substantial change to the nucleotide composition could potentially impact the structure and function of mRNA during its lifetime. Compared to an unmodified mRNA counterpart, a global m^1^Ψ-modified mRNA forms more thermally stable base pairs (9), has a different secondary structural composition (9), displays the greatest expression levels when the coding sequence and 3′-untranslated region are highly structured (9), is less susceptible to miRNA silencing (10), and maintains reasonably high translational fidelity to produce the desired protein product (11). However, little is known about how RNA structure and function is altered when the uracil content of a highly structured RNA element is replaced with m^1^Ψ.

**Figure 1:**
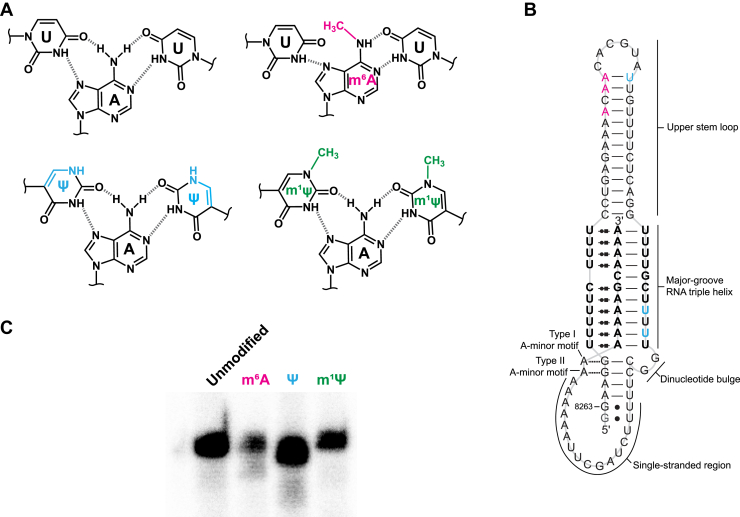
**Modified base triples and the MALAT1 triple helix.***A*, schematics of a canonical U•A-U base triple (*black*) and base triples with RNA base modifications: U•m^6^A-U (*magenta*), Ψ•A-Ψ (*blue*), and m^1^Ψ•A-m^1^Ψ (*green*). *B*, schematic of the MALAT1 triple helix with labels for specific regions and mapped modification sites (m^6^A sites in *magenta*, Ψ sites in *blue*) obtained from the m6A Atlas (m^6^A8287, m^6^A8290) (31), RMBase (m^6^A8287) (32), RMVar (m^6^A8289, m^6^A8290) (33), and DirectRMBD (Ψ8297, Ψ8317, Ψ8319) (34) databases. *C*, denaturing PAGE gel image of radiolabeled *in vitro*-transcribed RNAs: the unmodified MALAT1 triple helix and the MALAT1 triple helix globally modified with either m^6^A, Ψ, or m^1^Ψ. m^1^Ψ, *N*^1^-methylpseudouridine; m^6^A, *N*^6^-methyladenosine; Ψ, pseudouridine; MALAT1, metastasis-associated lung adenocarcinoma transcript 1.

To better understand the effects of global m^1^Ψ substitution on RNA structure and function, we selected the metastasis-associated lung adenocarcinoma transcript 1 (MALAT1) triple helix, an RNA stability element that contains multiple structural motifs: a major-groove RNA triple helix, type I and II A-minor interactions in the lower stem, a upper stem-loop, a dinucleotide bulge, and single-stranded RNA (Fig. 1*B*) (12). Inside cells, the triple helix presumably forms upon 3′-end maturation of MALAT1 *via* RNase P cleavage, whereby the A-rich tract and U-rich internal loop interact to form a predominantly U•A-U-rich triple helix (where • and – represent interactions along the Hoogsteen and Watson–Crick faces, respectively) (13, 14). Furthermore, this triple helix is recognized by methyltransferase-like protein 16 (METTL16), an m^6^A RNA methyltransferase, but METTL16 does not methylate the triple helix *in vitro* (15, 16, 17). Thus, the MALAT1 triple helix and METTL16 will allow us to probe how m^1^Ψ alters various RNA–RNA interactions (*e.g.*, Hoogsteen and Watson–Crick base pairs) as well as RNA–protein interactions. In addition to m^1^Ψ, we also examined the MALAT1 triple helix globally modified with *N*^6^-methyladenosine (m^6^A), a mRNA modification that has lower immunogenicity but produces no protein (1, 3), and pseudouridine (Ψ), a well-studied RNA modification that has also been pursued for use in therapeutic mRNAs (Fig. 1*A*) (5, 18, 19). m^6^A is likely to disrupt base triple formation because the *N*^6^ position mediates hydrogen bonds along both the Hoogsteen and Watson–Crick faces. However, Ψ and m^1^Ψ do not directly alter chemical groups that participate in Hoogsteen and Watson–Crick base pairs (Fig. 1*A*).

Herein, we show that the MALAT1 triple helix is generally stabilized when globally modified with either Ψ or m^1^Ψ, but not with m^6^A, based on UV thermal denaturation assays. All modifications disrupted binding between METTL16 and the globally modified MALAT1 triple helix, with the binding preference as follows: no modifications > m^6^A > m^1^Ψ > Ψ. Thus, this study shows that a globally modified RNA triple helix presents opportunities to manipulate its RNA structure and function for therapeutic purposes.

## Results and discussion

### Triple helix is generally stabilized when globally modified with Ψ or m^1^Ψ but not m^6^A

**Figure 2:**
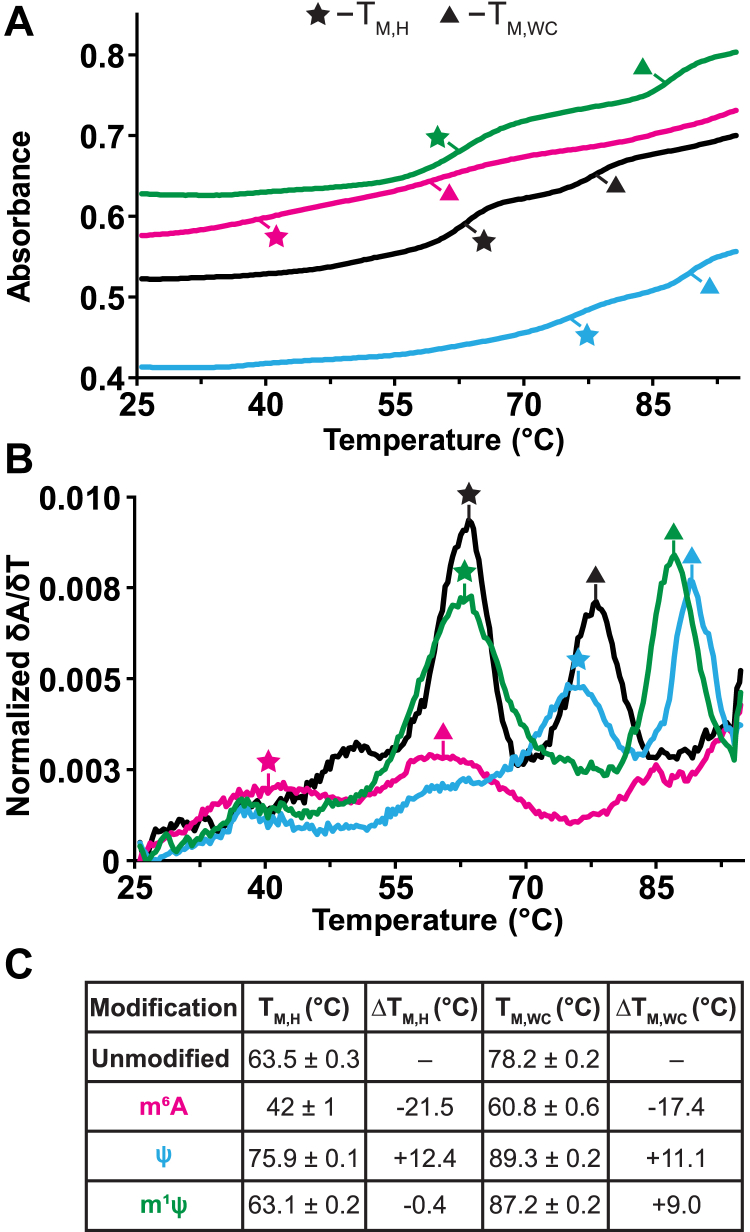
**UV thermal denaturation results for the MALAT1 triple helix globally modified with m**^**6**^**A, Ψ, or m**^**1**^**Ψ.***A* and *B*, representative plots of (*A*) absorbance *versus* and temperature and (*B*) of first derivative plots for the unmodified MALAT1 triple helix (*black*) and the MALAT1 triple helix globally modified with either m^6^A (*magenta*), Ψ (*blue*), or m^1^Ψ (*green*). T_M,H_ (*star*) and T_M,WC_ (*triangle*) are the melting temperatures for transitions representing denaturation along the Hoogsteen and Watson–Crick faces, respectively. *C*, melting temperatures for the MALAT1 triple helix with and without RNA modifications. T_M_ values represent an average ± standard deviation for three independent replicates. ΔT_M_ values were calculated as ΔT_M,modified RNA_ - ΔT_M,unmodified RNA_. m^1^Ψ, *N*^1^-methylpseudouridine; m^6^A, *N*^6^-methyladenosine; Ψ, pseudouridine; MALAT1, metastasis-associated lung adenocarcinoma transcript 1.

For this study, we generated the MALAT1 triple helix in its unmodified form and globally substituted with either m^6^A for A, Ψ for U, or m^1^Ψ for U. Full-length RNAs were obtained for all four RNAs (Fig. 1*C*). To quantitatively assess how these modifications perturb the thermostability of the MALAT1 triple helix, we employed UV thermal denaturation assays. This assay is an effective tool to examine stability because Hoogsteen interactions are a major contributor to the T_M,H_ peak at 63.5 °C and because Watson–Crick interactions (*i.e.*, upper and lower stems as well as what remains of the triple helix) are a major contributor to the T_M,WC_ peak at 78.2 °C (Fig. 2) (20, 21). Replacing all A nucleotides with m^6^A in the MALAT1 triple helix greatly destabilized both Hoogsteen and Watson–Crick base pairs by ∼20 °C, for the T_M,H_ and T_M,WC_ were respectively at 42 and 60.8 °C, indicating a greatly destabilized RNA structure (Fig. 2, magenta). In contrast, replacing all U nucleotides with Ψ in the MALAT1 triple helix stabilized all interactions compared to unmodified: ∼12 °C for the Hoogsteen melting transition and ∼11 °C for the Watson–Crick melting transition (Fig. 2, blue). Interestingly, replacing all U nucleotides with m^1^Ψ had a mild-to-no effect on the Hoogsteen interactions (−0.4 °C) yet stabilized all Watson–Crick interactions by 9 °C compared to the unmodified RNA counterpart (Fig. 2, green).

These trends involving Watson–Crick base pairing interactions (*i.e.*, T_M,WC_) are consistent with what has been reported in the literature: m^6^A destabilizes duplexes by 0.5 to 1.7 kcal/mol (22) due to the *syn* conformation of m^6^A when base paired with U, Ψ stabilizes duplexes by 1 to 1.7 kcal/mol (23) due to enhanced base stacking (24) and the ability of its *N*1 hydrogen to coordinate a water molecule (25), and m^1^Ψ-modified RNAs are more stable than unmodified RNAs in two independent UV thermal denaturation studies (7, 9). Although the effects of RNA modifications in double-stranded RNA have been studied, little is known about how these modifications affect the stability of triple-stranded RNA structures. For U•m^6^A-U base triples, it will be interesting to determine if there is a preference for m^6^A to adopt an *anti versus syn* conformation. Because we observed reduced T_M,H_ and T_M,WC_ values, it is possible that m^6^A may fluctuate between the two conformations. For Ψ, UV thermal denaturation assays show that a dA•Ψ-dA inverted motif (where a pyrimidine is located in the central position) is stabilized compared to that of dA•U-dA (26). In the context of an R•D-D triple helix, a single Ψ•A-T or Ψ•G-C did not significantly impact triple helix stability (27). Thus, our study provides early insights into how m^6^A, Ψ, and m^1^Ψ affect the stability of a U•A-U-rich triple helix: m^6^A is a destabilizer, Ψ is a stabilizer, and m^1^Ψ stabilizes the Watson–Crick, but not Hoogsteen, face of a major-groove triple helix.

### m^6^A, Ψ, and m^1^Ψ disrupt interactions between METTL16 and the globally modified MALAT1 triple helix

**Figure 3:**
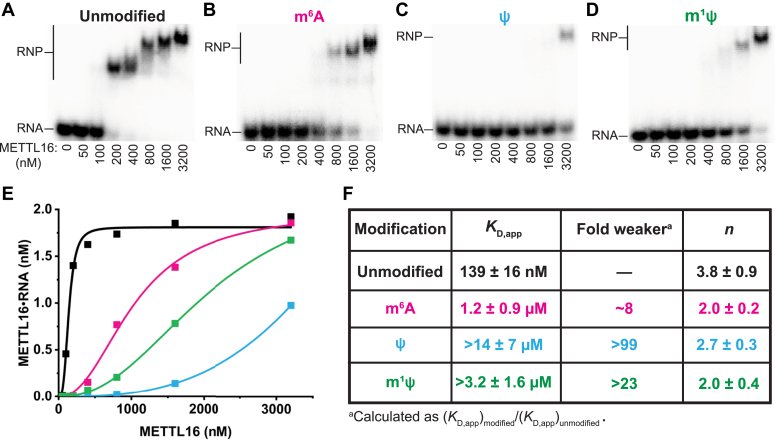
**Native gel-shift assays of METTL16 binding to the MALAT1 triple helix in the absence or presence of RNA modifications.***A*–*D*, representative gel images of METTL16 binding to the (*A*) unmodified MALAT1 triple helix, (*B*) m^6^A-modified MALAT1 triple helix, (*C*) Ψ-modified MALAT1 triple helix, and (*D*) m^1^Ψ-modified MALAT1 triple helix. *E*, representative binding plots of METTL16 for the unmodified MALAT1 triple helix (*black*), m^6^A-modified MALAT1 triple helix (*magenta*), Ψ-modified MALAT1 triple helix (*blue*), and m^1^Ψ-modified MALAT1 triple helix (*green*). *F*, measurements of apparent equilibrium dissociation constant (*K*_D,app_) between METTL16 and RNA with degrees of cooperativity (*n*). Data were fitted to the Hill equation (Equation 1). Binding reactions that did not reach saturation for an accurate measurement of *K*_D,app_ are denoted as larger than the extrapolated *K*_D,app_ value. Measurements are reported as average ± standard deviation of three independent replicates. m^1^Ψ, *N*^1^-methylpseudouridine; m^6^A, *N*^6^-methyladenosine; Ψ, pseudouridine; MALAT1, metastasis-associated lung adenocarcinoma transcript 1; METTL16, methyltransferase-like protein 16.

In addition to differentially affecting RNA–RNA interactions, RNA modifications also impact RNA–protein interactions. METTL16 recognizes the MALAT1 triple helix but does not methylate the RNA *in vitro* (15, 17). To determine the effect of m^6^A, Ψ, and m^1^Ψ modifications on METTL16•MALAT1 triple helix interactions, we utilized native electromobility shift assays. As observed previously for other RNA-binding partners of METTL16, multiple ribonucleoprotein (RNP) bands were observed for METTL16 binding to the unmodified MALAT1 triple helix despite the stoichiometry of RNA:protein being 1:1 (Fig. 3*A*) (17, 28, 29). METTL16•modified triple helix complexes did not exhibit such dramatic multiband shifts compared to unmodified (Fig. 3, *A*–*D*). Nonetheless, binding curves appeared to be cooperative, so we used the Hill equation to extrapolate the apparent equilibrium dissociation constants (*K*_D,app_) (Fig. 3*E*). Compared to unmodified RNA, METTL16 displayed weaker binding for all three globally modified forms of the MALAT1 triple helix, yet all four RNAs retained positive cooperativity, albeit slightly decreased for the modified RNAs (Fig. 3*F*). Surprisingly, despite destabilization of the global m^6^A-modified MALAT1 triple helix (Fig. 2), METTL16 preferred to bind to m^6^A-modified RNA over Ψ- or m^1^Ψ-modified RNA (Fig. 3). METTL16 bound to the global m^6^A-modified MALAT1 triple helix 8-fold weaker than unmodified, yet METTL16 binds ∼61-fold weaker to a single-m^6^A-methylated *versus* unmethylated U6 small nuclear RNA (snRNA) (17). The destabilized m^6^A-modified triple helix structure may allow room for METTL16 residues to form stabilizing interactions with nucleotides of the triple helix structure. Our UV thermal denaturation assays showed that global modification of either Ψ or m^1^Ψ generally stabilized the MALAT1 triple helix, yet both modified RNAs were poor binding partners of METTL16 with >20-fold higher *K*_D,app_ values (Fig. 3). A high-resolution structure of the METTL16•MALAT1 triple helix is not available, so the structural basis of how the three modified base triples would perturb binding to METTL16 is unclear. Possibilities include different degrees of RNA flexibility, different groove dimensions, altered hydrogen bond donor/acceptor pattern along the grooves, reduced hydrogen bonds, and/or steric hindrance due to methyl groups (30). According to various RNA modification databases, multiple m^6^A and Ψ sites have been detected in the MALAT1 triple helix and notably two occur in the major-groove triple helix (Fig. 1*B*) (31, 32, 33, 34). Although site-specific modifications are less extreme than global modifications, our results suggest that site-specific m^6^A and Ψ modifications may represent ‘structural switches’ that alter the half-life of MALAT1 and/or METTL16 binding to the MALAT1 triple helix inside cells (35, 35). Thus, it will be interesting to uncover the functional significance of m^6^A and Ψ marks with respect to the METTL16•MALAT1 triple helix complex.

Because naturally occurring RNAs rarely exist in a globally modified state, little is known about the impact of global RNA modifications on protein interactions. However, weaker RNA•protein interactions appear to be a general trend unless the function of the protein is to recognize RNA modifications, such as the class of ‘reader’ proteins. For example, human Pumilio homolog 2 binds to a specific sequence in single-stranded RNA. Single-stranded RNAs substituted with 1 to 3 m^6^A or Ψ residues weakened Pumilio homolog 2 binding to RNA from ∼2- to 38-fold for m^6^A and from ∼2- to 380-fold for Ψ, all due to faster dissociation rates compared to unmodified RNA (36). Weaker binding has also been observed for the protein muscleblind-like 1 interacting with Ψ-modified CCUG/CUG repeats, albeit context-dependent: 6 Ψ in a CCUG repeat weakened binding only ∼1.5-fold but 2 or 4 Ψ in CUG repeats weakened binding more than 20-fold relative to unmodified RNA (37). This study suggested that the Ψ-modified CUG repeat RNA is stabilized by greater base-stacking interactions, and the rigidity of RNA induces suboptimal conformations for muscleblind-like 1 to bind (37). It is important to note that not all modified RNAs disrupt interactions with proteins. Reader proteins, such as the m^6^A reader YTH domain-containing protein 1, bind with a *K*_D_ of 300 nM to an RNA with a single m^6^A site, but no binding is detected for the unmodified counterpart (38). Similarly, a larger fraction of yeast pre-mRNA processing ATP-dependent RNA helicase Prp5 co-immunoprecipitates with Ψ-modified U2 snRNA than the Ψ-deficient counterpart, and a global Ψ-modified U2 snRNA enhanced the ATPase activity of Prp5 by 3-fold compared to unmodified RNA (39). More studies will need to be performed to fully understand how a single *versus* global RNA modifications will affect an RNP complex.

With mRNA therapeutics greatly benefiting from the incorporation of naturally occurring modifications, an increasing number of RNA-centric systems are being examined to determine if RNA modifications are advantageous. For CRISPR/Cas9 as a genome-editing tool, cleavage reactions performed with tracrRNA globally modified with m^6^A, m^5^C, or Ψ generally showed the following trends with respect to strand cleavage: unmodified ≈ m^5^C > Ψ >> m^6^A (40). When the sgRNA was globally modified, then the general trend for strand cleavage changed to unmodified > m^6^A ≈ m^5^C >> Ψ (40). Notably, these trends did depend on the percent of modification so their effects are tunable (40). In theory, the MALAT1 triple helix is a stability element that could be used to replace the polyA tail of therapeutic mRNAs because the triple helix is effective at stabilizing reporter mRNAs (β-globin and GFP), L1 retrotransposon, and telomerase expressed from plasmids (20, 41, 42, 43). However, the MALAT1 triple helix is derived from a cancer-promoting long-noncoding RNA (44), so it may be risky to use until it is clearly established that the MALAT1 triple helix does not have any direct role in promoting cancer, metastasis, or possibly viral infections (45, 46, 47, 48). Additionally, there are other effective means to stabilize therapeutic mRNAs, such as IRES and circularization (49, 50). Another consideration is whether the immune system can sense invading triple-stranded RNA structures, which is unknown to the best of our knowledge. However, cellular receptors, such as toll-like receptor 3, and cytoplasmic receptors, such as MDA5 and LGP2, interact with double-stranded RNA *via* the sugar-phosphate backbones and minor and major grooves (51, 52, 53). Considering helix diameter and minor-groove dimensions are comparable between RNA double helices and major-groove RNA triple helices (54), then receptors that recognize double-stranded RNA may be able to recognize triple-stranded RNAs. However, therapeutic triple helix RNA structures may evade detection if the third strand is globally replaced with RNA modifications, thereby disrupting receptor interactions with the RNA triple helix (8).

## Experimental procedures

### Preparation of RNAs and METTL16

To prepare the MALAT1 RNA triple helix (sequence shown in Fig. 1*B*), oligonucleotides were synthesized by Sigma-Aldrich, and then DNA templates were generated using PCR. *In vitro* transcription reactions were completed as previously described (55). Briefly, reactions used homemade T7 RNA polymerase and included 2 mM each of ATP, CTP, GTP, and UTP (MilliporeSigma) for unmodified RNA and then substitution of the appropriate unmodified NTP for one of the modified nucleotides, m^6^ATP, ΨTP, and m^1^-ΨTP (Jena Biosciences), for modified RNAs. After NAP10 column purification, RNA was quantified using a NanoDrop One^C^ instrument (Thermo Fisher Scientific), and the yields were 40 to 60% less than unmodified RNA. RNAs were 5′-[^32^P]-radiolabeled by first using alkaline phosphatase (New England Biolabs) to remove the 5′-triphosphate group and then using γ-[^32^P]ATP (PerkinElmer) and T4 PNK (New England Biolabs) to radiolabel RNA per the manufacturer’s protocols. Unreacted γ-[^32^P]ATP was removed by passing the reaction mixture through a Microspin G-25 column (Cytiva). To verify full-length RNAs, RNAs were resolved on an 8% denaturing PAGE, gel was exposed overnight to a Phosphor screen, and the Phosphor screen was then scanned using an Amersham Typhoon IP Phosphorimager (GE Healthcare).

Full-length human METTL16 with a C-terminal His_6_-tag and TEV protease cleavage site was expressed in BL21 Gold *E. coli* (Agilent) and purified using nickel-affinity and heparin-affinity chromatography as previously described (17). Fraction of binding-competent METTL16 was determined as previously described (56). Briefly, fraction of binding-competent METTL16 was calculated by observing binding between increasing amounts of METTL16 (125–2000 nM) and 5′-[^32^P]-radiolabeled unmodified MALAT1 triple helix (500 nM), where the concentration of the MALAT1 triple helix was >2-fold greater than the measured *K*_D_. The amount of active protein was calculated by first plotting fraction bound *versus* protein:RNA ratio and then using the breakpoint ratio, 1.875:1, to determine the percent of active protein. Protein concentration calculated from UV absorbance (A_280_) was then adjusted based on the protein being 53% active. The active site concentration was used for the native gel-shift assays.

### UV thermal denaturation assays

RNA samples (∼0.2–0.3 μM) were diluted in 1× melting buffer (25 mM sodium cacodylate pH 7, 50 mM KCl, and 1 mM MgCl_2_) and then transferred to a stoppered 1-cm quartz cuvette. Samples were inserted into an Agilent Cary 3500 multicell peltier UV-vis spectrophotometer and then folded *via* rapid heating (from room temperature to 95 °C at 5 °C/min) and cooling (from 95 °C to 25 °C at 5 °C/min) immediately before the absorbance (A_260_), and temperature data were collected for the denaturation step (from 25 °C to 95 °C at 0.8 °C/min with A_260_ values collected at 0.3 °C intervals). Individual melting profiles were processed as follows: subtract buffer background from absorbance values of RNA, calculate first derivative of absorbance with respect to temperature (δA/δT), smooth the δA/δT values across a 1.2 °C window using the Savitzky–Golay method, plot values *versus* temperature, and extrapolate melting temperatures (T_M_) from the peak maxima of plot. T_M_ values are an average of three independent measurements and error represents standard deviation.

### Native gel-shift assays

RNAs were 5′-[^32^P]-radiolabeled by first using alkaline phosphatase (New England Biolabs) to remove the 5′-triphosphate group and then using γ-[^32^P]ATP (PerkinElmer) and T4 PNK (New England Biolabs) to radiolabel RNA per the manufacturer’s protocols. Unreacted γ-[^32^P]ATP was removed by passing the reaction mixture through a Microspin G-25 column (Cytiva). 5′-[^32^P]-labeled RNA was folded by heating to 95 °C for 3 min, snap cooling on ice for 5 min, and allowing to equilibrate at room temperature for 30 min. Increasing concentrations of binding-competent METTL16 (50–3200 nM) were titrated into the binding solution, containing 2 nM RNA, 25 mM Tris-HCl pH 7.5 at 25 °C, 150 mM KCl, 1 mM MgCl_2_, 1 mM DTT, 0.5 mg/ml tRNA, and 7.5% glycerol at room temperature for 30 min. Binding reactions were resolved *via* 5% native PAGE for 2 h at 135 V, gels were exposed to a Phosphor screen and screens were imaged using an Amersham Typhoon (GE Healthcare). Band densities for free RNA and RNP were quantified using ImageQuant software (GE Healthcare); bands used for RNP correspond to labels in Figure 3, *A*–*D*. To determine the equilibrium dissociation constants (*K*_D_), plots of complex formation *versus* the concentration of METTL16 were fit to the Hill equation (Equation 1) using Origin software.

(1) $$y=\frac{R_{\text{total}\;}\times \;{P_{\text{total}}}^{n}}{{K_{D,\text{app}}}^{n}+{{\;P}_{\text{total}}}^{n}}$$

In Equation 1, *y* refers to the concentration of METTL16•RNA complex, *R* refers to the concentration of RNA, *P* refers to the binding-competent concentration of METTL16, *K*_D,app_ refers to the apparent dissociation constant, and *n* refers to the degree of cooperativity.

## Data availability

All data are contained within the article.

## Conflict of interest

The authors declare that they have no conflicts of interest with the contents of this article.
